# A New Method to Assist Decision-Making of Water Environmental Emergency in Expressway Region

**DOI:** 10.3390/ijerph191610043

**Published:** 2022-08-15

**Authors:** Qing’e Wang, Mengmeng Su, Lei Zeng, Huihua Chen

**Affiliations:** 1 School of Civil Engineering, Central South University, Changsha 410075, China; 2 Guangzhou Expressway Co., Ltd., Guangzhou 510288, China

**Keywords:** emergency decision-making, water environment, scenario retrieval, CBR, emergency classification

## Abstract

Water environmental emergency (WEE) in expressway region is a special kind of risk event with several characteristics, such as rarity, unconventionality, and harmfulness. The emergency decision-making (EDM) features, procedures, and methods are considerably different from the general decision-making problems. EDM quality is directly related to the timely implementation of a reasonable emergency plan. Therefore, methods should be developed to respond to emergencies immediately and scientifically and minimize the damage to water environment. This work introduces risk source identification and emergency classification and develops an emergency decision model based on scenario retrieval and case-based reasoning, according to the existing EDM model and characteristics of WEE in expressway region. The proposed method is validated through case analysis of Daguang expressway in China. This method provides an effective solution for EDM of WEEs in expressway region. The emergency measures can be implemented quickly and effectively after the occurrence of water environmental emergencies to control pollution events, provide scientific and feasible action guides for emergency processes, and enrich the case base of decision-making systems.

## 1. Introduction

During expressway construction and operation, many pollutants, such as solids, nutrients and heavy metals will be produced [1] and the surface runoff will have a negative impact on the water environment [2]. Also, most expressways are linear, and crossing some important rivers, tourist sites, and water conservation areas is inevitable. Thus, during construction and operation periods, the effect of road hazardous chemical transportation accidents on the water environment in expressway region should be given great attention. These water environmental emergencies (WEE) exhibit several characteristics, such as infrequency, timeliness and dynamics. The major water pollution emergencies in expressway region have severely damaged the water environment and caused serious pollution to drinking water sources, thereby posing a direct threat to people’s health and life [3]. Therefore, it is of great practical significance to study how to make decisions and take measures to effectively deal with water environment emergencies to avoid water environment pollution and other losses [4,5].

The pollution sources of water pollution emergencies are complex, it is essential to select an appropriate disposal technology for a specific pollution scenario. Studies on water pollution accidents are mainly carried out from two aspects: simulating the spread of pollutants and assessment. He et al. developed an integrated water pollution management information system to simulate the transfer and diffusion of accidental pollutants in the river and quickly identify the affected area and how it will change over time within a few minutes of an accident occurring [6]. Rui et al. proposed an emergency response system with a hydraulic/water-quality model [7]. Ding et al. combined with two-dimensional water quality model and GIS to simulate accidents and developed an accident trend prediction system [8]. However, due to the uncertainty and complexity of water pollution accidents, most water quality models used to simulate pollution accidents have complex structures and multiple parameters. This limits the emergency decision-making mode of water pollution accidents. The assessment method can provide a targeted reference or guidance for emergency control decision makers. Long et al. proposed an integrated assessment method by incorporating an improved technique for order preference by similarity to an ideal solution. Emergency response plan optimization is the key to handling accidents [9]. Qu et al. developed a novel two-stage evaluation system to determine the optimal emergency treatment technology scheme [10], later proposed a three-stage technical screening and evaluation tool to determine the optimal technique scheme. Existing studies focus more on methods of calculating and evaluating, but fail to take identification and classification of emergencies into account in the process of emergency management.

To deal with emergencies effectively, it is necessary to solve decision-making problems in the process of emergency management to formulate emergency plans. Decision-making consists of three main components, namely, decision makers, decision tools, and techniques for selecting the best alternative [11]. The ultimate goal is to obtain an emergency decision scheme with group satisfaction and low risk degree [12]. From the temporal perspective, the emergency decision-making (EDM) work can be divided into warning precaution, crisis identification and isolation, integrated response, and disposal measures [13]. Prediagnosis of emergency response potential and decision support during rescue operations are essential for improving emergency response capability [14]. Extensive research has been carried out on methods and applications of EDM. Complex scientific information should help emergency managers effectively prepare for disasters [15]. Different EDMs can be developed by combining and integrating various methods. Kou et al. integrated fuzzy logic, questionnaires, Delphi method, and multi-criteria decision-making method, and proposed an efficient disaster assessment expert system [15]. Tang et al. proposed a novel emergency response plan structure that considers emergency command operation requirements [16]. Zhou et al. elaborated the decision-making method of Bayesian network, game theory, fuzzy theory, and situational evolution and developed a natural disaster EDM system [17]. Sierra et al. used Bayesian networks to support decision-making in environmental problems [18]. Different decision-making methods are also applied to specific emergency management of various emergencies. Yuan et al. used the Fault Tree Analysis to study the influential factors in the emergency process of fire accident for oil-gas storage and transportation [19] and determined four aspects, namely, human, material, environment, and management, as the effects in the emergency process of fire accidents for oil and gas storage and transportation systems [20]. Alvear et al. proposed a decision support system (DSS) for emergency management of road tunnels [21]. Ding et al. proposed an EDM method with a hybrid use of dynamic network game technology, Bayesian analysis, and multi-attribute utility theory [22]. There are plenty of studies about emergency decision making. Bayesian networks, scenario analysis and CBR have been applied in EDM. But EDM research on some specific types of events are still scarce.

Water environmental emergencies in expressway region is defined in this research as the event that the risk source of expressway deviates from normal condition due to mechanical failure, human factors, poor management and environmental uncertainty, which causes sudden discharge of pollutants and poses a threat to the water environment of expressway region. The existing research of water environmental pollution accidents is mainly on the simulation and assessment, and the ecological risk associated with a range of human activities is considered to be a major cause of water environmental pollution [23], and also, consequences of water pollution accidents affect human health [24]. Adequate emergency measures can avoid or reduce water environmental pollution caused by emergencies to the greatest extent [25], and realize effective risk management [26]. Research on emergency management for water environment emergency the mainly focuses on the early warning of sudden environmental pollution accidents [27,28]. To make up for the lack of research on water environmental emergencies in expressway region and assist decision-making for managers to deal with them, emergency decision methods need to be studied. WEE in expressway region are typical and special and need an individual study on the event disposal. Therefore, the identification and classification of risk sources is also very important in the treatment of water pollution emergencies. Such water pollution emergencies require a more comprehensive case base, which can quickly and accurately select the best plan according to the actual emergency events and assist the managers in making decisions. Moreover, The EDM of water environmental emergency in expressway region has some similarities between general EDM, but because of the infrequency, timeliness and complex risk resources, there is a need of special research on the WEE. Based on the background of WEE in expressway region, we attempt to introduce risk source identification and emergency classification into the EDM process and propose a EDM model combining scenario analysis and CBR research methods. This study aims to build a model for emergency decision-making and verify the feasibility of the model through specific case analysis, to help with water environment emergency decision making and provide reference for the development of decision support system.

## 2. Research Methods

### 2.1. Introduction of Research Methods

Prior research has performed substantial work about simulation of the spread of pollutants, assessment for emergency response plan, or methods and applications of EDM. However, former decision-making methodologies applied to other research topics are not entirely applicable to EDM research on WEE in expressway region. In addition, few studies consider the relationship between emergency classification and EDM. Therefore, this study proposes a model and introduces risk source identification and emergency classification into this model. Also, this study build a case base and conduct case studies to maintain the system learning and update. The proposed method of this study, assisting decision-making of water environmental emergency in expressway region, mainly involve scenario analysis and CBR. This work describes methods in the WEE decision-making process for addressing unconventionality and scenario-dependency of emergencies to bridge this gap.

### 2.2. Scenario Analysis

The description of a possible future and its corresponding path constitutes a scenario [29]. Scenarios, as an important technique of studying the future, have been used by government agencies, corporate industries and military unites as powerful tools to help in the decision-making of uncertainty for a long time [30]. Scenario analysis is an effective way to formulate strategic planning towards future [31,32]. When faced with high levels of uncertainty and complexity, scenarios analysis can help make decisions that are difficult to obtain through traditional predictions [33]. Basing on similar existing scenarios, it becomes more efficient to newly describe target scenarios. Scenario retrieval method using differential information between two scenarios was proposed, which enables the detection of similar scenarios for a given scenario [34].

Scenario analysis was first applied to military and has now been successfully applied in various areas, for example, environment [35,36,37,38,39,40], conomics [41,42], emerging technology [43], infrastructure [44] and construction project [45,46]. In recent years, scenario analysis has also been widely used in disaster risk and emergency management. Li et al. proposed that different assessment models and methods are needed to match with different stages in unconventional emergency disposals [47]. Unconventional emergency management and decision-making frameworks were developed on the basis of “scenario-response” [48,49,50]. Lv et al. studied emergency evacuation management and risk analysis under multiple uncertainties with a scenario-based modeling [51]. Davies et al. used a scenario-based approach to co-produce and integrate hazard and risk knowledge towards disaster resilience [52].

The WEE decision-making in expressway region is characterized by uncertainty and information asymmetry. Bayesian network is an effective theoretical model of uncertain knowledge representation and reasoning. This network can deduct with incomplete, imprecise, or indeterminate messages to support modeling and calculating complex decision-making problems. The network characteristics determine the applicability to solve such problems. Compared to the entire emergency, a scenario is relatively stable during a certain period of time and is not significantly rare. Therefore, this study uses scenario analysis methods, combined with Bayesian networks, to derive scenarios and obtain feature values to be prepared for CBR.

### 2.3. Case-Based Reasoning

Case-based reasoning (CBR) provides a new way to address the problem in the artificial intelligence domain. This approach refers to the mode of thinking and dealing with problems by referring to human beings. When encountering a problem that needs to be solved, CBR will first look for similar events in the past, then repair solutions of such time, and finally form a solution to the problem. CBR is based on approximate reasoning and knowledge [53,54]. Retrieval is a key phase in CBR because it lays the foundation for the overall effectiveness of CBR systems [55]. Bottlenecks of CBR method application are knowledge acquisition and case representation and need to be handled [56]. CBR can be translated into mature practical applications [57,58]. The research proves the evolution rules of CBR in drilling engineering and data collection of crossed applications. The popularity of CBR applications in CM research [59], such as building safety [60], and bidding risk [61], is increasing. 

EDM, the main field of CBR technology, has received extensive attention from scholars, such as: (1) an emergency procurement DSS (EPDSS) on the basis of key technologies, including case representation, case retrieval algorithm, and hybrid reasoning mechanism [62]; (2) a method for emergency resource demand prediction using CBR [63]; (3) a marine oil spoil emergency response decision-making system with CBR technology [64]; (4) analysis of the response to risks connected with urban water supply network in the CBR method [65]; and (5) the use of CBR for contingency decision support for the United States Air Force [66].

## 3. Proposed Conceptual Model and Methods

### 3.1. Introduction of the Conceptual Model

Zhou et al. [17] explain that decision-making is a process of selecting an optimal scheme in various alternatives. The EDM process includes six stages, namely, problem definition, goal setting, project design, project selection, organization implementation, and feedback modification. The CBR approach is an analogical reasoning method. This method includes the following steps [64]: demonstration of cases to be solved, index search, and updated solutions of similar cases. Yuan and Li [49] research emergency management with scenario analysis and CBR. This study combines SR with CBR in EDM based on the work of Yuan and Li [49], according to the above-mentioned research models. Then, a method of generating an EDM plan is developed for water environmental emergencies.

The particularity of water environmental emergencies in expressway region exhibits the typical characteristics of risk decision-making. Defining risk sources of water environmental emergencies is the premise and basic work for EDM and emergency management of such emergencies. Therefore, this study proposes that risk sources should be first identified based on previous research. Taking appropriate emergency methods and measures for emergencies classification is a common experience of countries in the world when dealing with emergencies. Different types and levels of emergencies directly determine the main body, measures and resources that must be mobilized in response to emergencies. Scientific and accurate classification can avoid insufficient reaction and overreaction. In the case study and storing process, classification can help the management of the case base and ensure the accuracy of the next case reasoning. Therefore, this study introduces classification measures for emergencies. 

**Figure 1:**
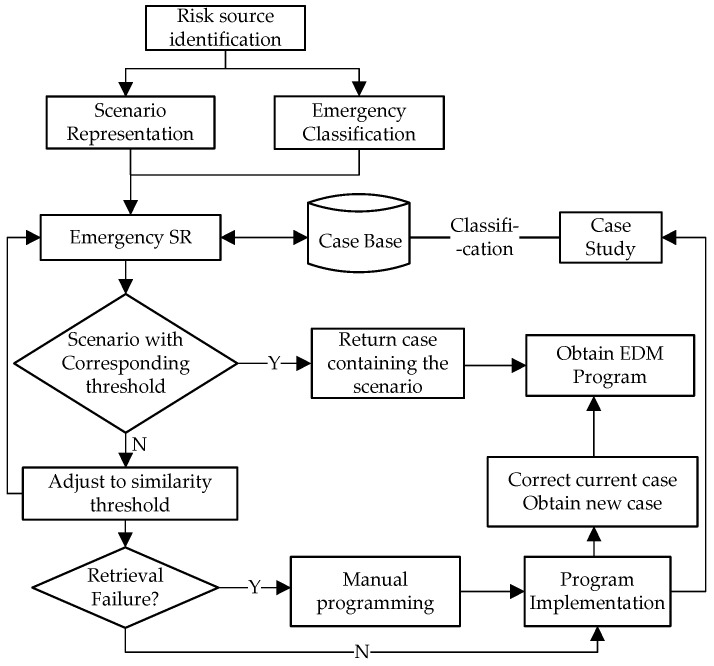
EDM conceptual model for WEE based on SR and CBR.

As shown in Figure 1, the conceptual model proposed in this study includes the following processes: 1. In the risk identification stage, risk sources and their potential impacts are determined through expert interviews and questionnaires. 2. In the emergency classification stage, the dynamic fuzzy comprehensive classification algorithm is used to classify the accident, and the cluster analysis and minimum distance method are further used to classify the emergency. 3. In the scenario representation stage, the feature values of WEE were obtained by combining scene analysis and Bayesian network method. 4. In the CBR stage, the nearest-neighbor method is selected to retrieve similar scenarios from case base, and the emergency plan of the similar case scenario forms the basis of making EDM plan for WEE.

### 3.2. Detailed Description of the Methods in the Model

#### 3.2.1. Risk Source Identification

The source of hazard is the risk source of environmental pollution, which is a prerequisite for the occurrence of pollution accidents. Understanding the risk source of water environmental emergencies objectively and accurately in expressway region not only contributes to the pre-existing control of risks but also helps to improve the efficiency of emergency rescue and the targeting of emergency response plans. This approach can minimize various effects, such as casualties, traffic disruption, and property damage. It can also narrow the scope of the disaster, and mitigate the negative effects on society.

In this work, the risk source of water environment in expressway region is defined as not only the toxic and harmful substances that can pollute the water quality but also the leakage, diffusion, and migration of the substances and environmental factors that can affect an area within 200 m of each side of the centerline of expressway (or 100 m upstream or 100 m downstream of the centerline when crossing the water) during the life of the expressway project. For example, in the Jinxiu event which caused the leakage of dangerous substances, a car carrying arsenic fell into the river, a large amount of arsenic was scattered on the riverside slope, partially scattered into the river, causing the pollution of water, riverside slope and vegetation.

#### 3.2.2. Emergency Classification

Water environmental emergencies exhibit uncertainty and diffusivity. The toxicity and total quantity of dangerous substances are partially determined for the maximum credible accident in expressway. The classification index presents fuzziness and imprecision, and the material leakage is partially determined. Hence, the classification index of the maximum trusted accident demonstrates fuzziness and imprecision. Therefore, the selection of the classification method for WEE should present good elasticity and flexibility.

The introduction of the triangular fuzzy number method to represent the weights among classification indexes can accurately reflect the uncertainty and precision. The optimal scheme of fuzzy comprehensive evaluation is obtained according to the Kerre method. And the pessimistic decision-making principle is adopted to select the highly pessimistic value from the fuzzy value of the optimal scheme as the definite final value, which accords with the principle of minimum repentance value of the psychological behavior. The corresponding classification table determines the final level of the WEE. Then, multiple water environmental emergencies that are at the same level were clustered by the shortest distance method and subdivided.

#### 3.2.3. Scenario Definition and Representation

**Figure 2:**
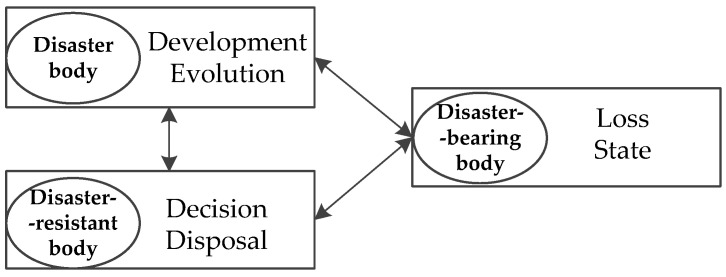
3D structure of the elements of water environmental emergency scenario.

A specific scenario of a WEE can be described from three dimensions, namely, disaster body, disaster-resistant body, and disaster-bearing body. The relationships between these three dimensions (3D) are shown in Figure 2.

The three dimensions can be expressed as shown in Formula (1):

(1) $$S_{i}\left(x_{i},y_{i},z_{i}\right)\;i=1,2,3,\ldots ,m,$$

*S* is the WEE scenario, *x* is disaster body dimension, *y* is disaster-resistant body dimension, *z* is disaster-bearing body dimension, and *m* is the quantity of critical scenarios during the evolution. The variables *m* and *i* can be either the same or different.

**Figure 3:**
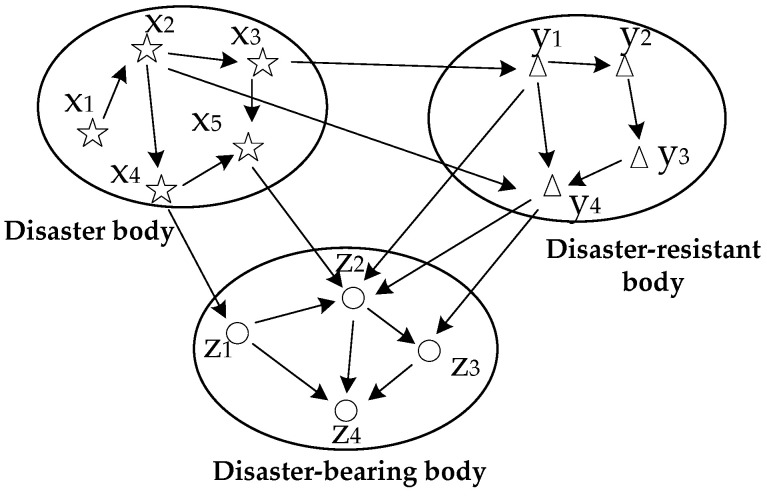
Relationship of scenario elements of water environmental emergencies.

The relationships between scenario elements are shown in Figure 3, and the arrows indicate influential relationships. Two types of relationships exist between the elements at one time: (1) The relationships between elements in one dimension. For example, emergency resource arrival time presents an effect on the implementation of emergency measures. These relationships belong to disaster-resistant body dimension. (2) The relationships between elements in different dimensions. The pollutant leakage quantity, as a disaster body, affects the degree to which water is contaminated (disaster-bearing body).

#### 3.2.4. Bayesian Network Construction and Deduction

**Figure 4:**
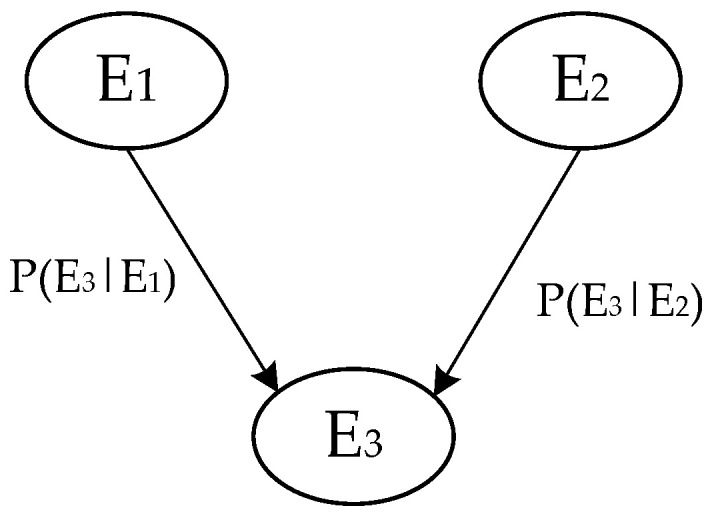
Bayesian network.

The Bayesian network of a scenario is shown in Figure 4.

In Figure 4, nodes *E*_1_, *E*_2_, and *E*_3_ represent different variables of the scenario. The joint probability of the variables in Figure 4 can be expressed as follows:

(2) $$P\left(E_{1},E_{2},E_{3}\right)=\prod_{i=1}^{3}p\left(Ei|Pa\left(Ei\right)\right)=P\left(E_{3}|E_{1}\right)P\left(E_{3}|E_{2}\right),$$

Figure 3 provides a prototype for the Bayesian network structure of a water environmental emergency scenario from the above-mentioned analysis. Then, Bayesian network node variables along with their relationships and the conditional probability of assignment nodes are determined, as studied by Yuan [67].

**Figure 5:**
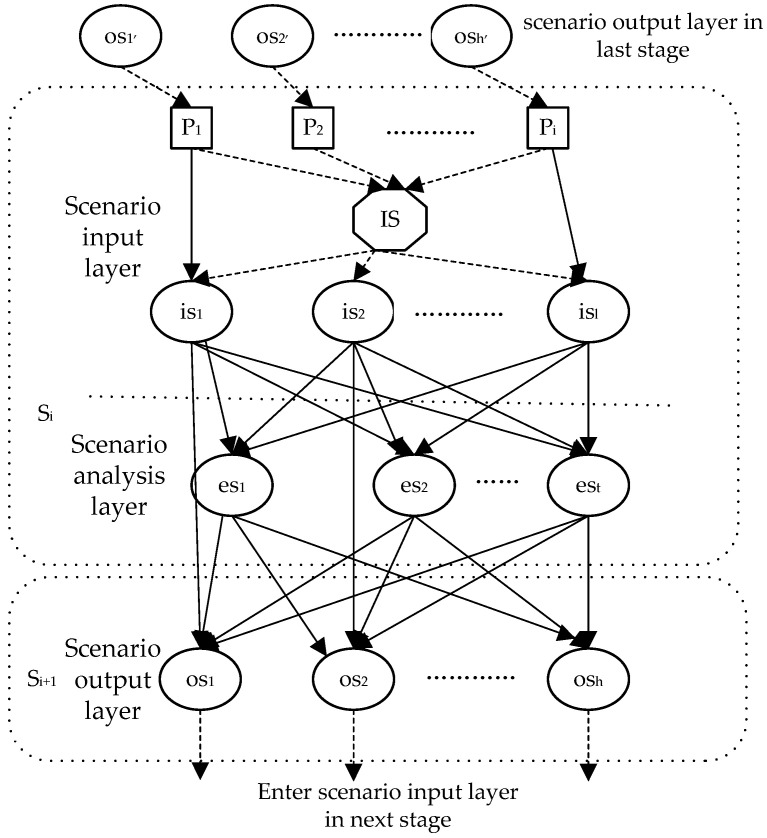
Deduction network of WEE scenarios.

The joint probability formula of a Bayesian network can be applied to deduce state probabilities of node variables in the scenario output layer after assigning prior or conditional probabilities of network node variables, as shown in Figure 5.

After the state probabilities are obtained, with other scenario information, the scenario output layer can be the next scenario analysis layer and so on. The deduction process can be completed until it enters into the final scenario.

#### 3.2.5. CBR

The WEE CBR is built on SR, and the goal is to retrieve scenarios with similar features. The nearest-neighbor method retrieval algorithm is adopted according to the features and contents of WEE cases. A threshold value u is required to determine whether the case scenario in the corresponding case base is similar to the scenario to be solved. If multiple cases exhibit similarity greater than this threshold, the case scenario whose similarity value is closest to one is the most valuable case retrieved.

The realization of the retrieval algorithm involves the following four steps: determination of retrieval feature attributes, evaluation of weights of retrieval feature attributes, calculation of attribute similarity, and calculation of global similarity [68].

#### 3.2.6. Case Learning and Storing

After implementing the obtained emergency response plan, the scenario and emergency response plan can be supplemented with the necessary information. And relevant feedback information can be recorded. The learning rule is used to determine whether to add the case to case base for implementing the self-learning function of the CBR system. No in-depth research of the case study process was conducted in this work. Below is a brief introduction:

Machine learning is a branch of the artificial intelligence field. Any research on learning algorithms through data training belongs to machine learning, including several technologies that have been developed for many years, such as KNN, SVM, linear regression, K-means, random forest, PCA, and ANN. This work proposes the cluster analysis method to classify emergencies, which also means that this study is applicable to unsupervised learning algorithms. Hidden information in data sets can be extracted using clustering results to classify and predict future data. However, the actual system contains a considerable amount of case data, and the traditional machine learning algorithm seems to be unable to deal with big data.

Hinton made a breakthrough in the artificial neural network [68], thereby giving the neural network a “depth”, set off a wave of deep learning, and provided an effective solution for big data. The main differences between deep learning and traditional machine learning lies are as follows:

Traditional machine learning needs to be characterized by industry experts and then manually coded. The model rules are relatively simple. Meanwhile, a deep learning algorithm attempts to learn features from the data itself, thereby making the internal rules of deep learning model difficult to understand. In addition, deep learning heavily relies on high-end hardware facilities and long training time due to the substantial amount of computation. The top-level deep learning algorithm ResNet takes two weeks to train. Adopting traditional machine learning method at the beginning is feasible during the expressway WEE since ordinary machine learning exhibits no particularly high hardware requirement, and the training usually takes just a few seconds to hours. However, the application of deep learning algorithm is inevitable with the increase of the number of samples and data.

## 4. Validation of Method with a Case Study

### 4.1. Description of Case Study Scenario

Concentrating on one case allows us to collect substantial data, which is important to analyze a phenomenon that is yet to be fully understood [69]. The “4.25 event” of Daguang(DG) expressway is chosen as a case study scenario. A tanker carrying 16.13 tons of petroleum rolled over near the Liuxi River water source on DG expressway. The petroleum leaked into the river, and the tanker caught fire. During handling of the accident, a rear-end collision happened to two tank trucks carrying hazardous chemical behind the former accident. One of the trucks carrying white oil and solvent naphtha burned and exploded, and the other one carrying styrene got fired and burned. Moreover, petroleum and other toxic and harmful substances leaked, then spread through the water, thereby causing serious river pollution. The entire WEE was called the “4.25 event”. This incident is a typical WEE with serious hazard, huge impact, and sufficient data. Therefore, the incident is selected as a research case.

### 4.2. Data Collection and Analysis of Indicators

This study got the identification of potential risk events and the most credible accident grading indicators through questionnaires, interviews and expert scoring, providing a basis for case studies. After the questionnaire was collected, SPSS 17.0 statistical software was used and the Mean and standard deviation (S.D) was obtained for data analysis. When Mean > 3, the result was chosen, which means that the respondents think it’s important. Reliability is an important indicator to measure the quality of a set of data. It can test the stability or reliability of the questionnaire and the inherent consistency of the survey results. Generally, coefficient value of Cranbach α is between 0 and 1. When the coefficient does not exceed 0.6, the internal consistency is insufficient; when it reaches 0.7–0.8, the scale has considerable reliability; when it reaches 0.8–0.9, the reliability of the scale is great. According to the investigation results, events that may pollute the water environment (potential risk events) are presented in Table 1.

From Table 1, we can find that the potential risk events of water environment in expressway region covers design problems of the infrastructures, improper construction materials and methods, leakage from machine, equipment and vehicle, improper storage and the lack of safety awareness.

From Table 2, the Cranbach α value of this part is 0.889, and the standardized Cranbach α value of the evaluated items is 0.879. According to the Cranbach α, the reliability of the survey scale is great. Before the questionnaire, unimportant risk events had been eliminated through experts’ interviews. According to the questionnaire, the Mean of each risk event is above 4 (except case 9 with Mean being 3.99), which indicates that these risk events are more likely to pollute the water environment. The results of the study on factors affecting the consequences of the most credible accidents and the grading indicators are shown in Table 3.

From Table 3, after further expert scoring, it’s found that the risk events that have a greater impact on the water environment include leakage substances, treatment of leakage substances, total amount of leakage substances, emergency time, emergency duration, emergency form, leakage location, water flow velocity, wind speed.

From Table 4, the Cronbach α value of this survey is 0.747, and the standardized Cronbach α value of the evaluated item is 0.747. High reliability of the survey scale is proved in terms of the Cronbach α value. The nine grading indicators were scored and evaluated by experts. Except for the “wind speed” scoring 3.88 which meant the experts had minor differences, the Means of other eight grading indicators were all above “4” points, which meant they were crucial.

### 4.3. Risk Source Identification

The DG expressway project passes through headwater protection areas containing the Liuxi River reservoir, Huanglong Belt reservoir, and Liuxi River, which are surrounded by rich water system, considerable mountains with slope steep, and abundant rainfall. The effect of the project on headwater protection areas can be divided into two aspects. The first one is the potential threat to the water quality of vehicles transporting hazardous goods during the operation period. The second one is the potential risk that remained due to unmet environmental protection design requirements and standards in the design period. Risk source identification is mainly carried out by expert interviews and questionnaire surveys according to the actual situation of DG. Based on the work in 3.6, the risk source identification and their potential effects on the water environment in DG expressway region combined with the actual situation of the construction environment are shown in Table 5.

Historical information, relevant engineering, video surveillance, road administration, maintenance personnel daily inspection, expressway monitoring system, alarm and other means are helpful to timely discover emergencies on the spot.

### 4.4. Emergency Classification

The maximum credible accident of water environment in expressway region refers to emergencies, such as leakage of dangerous substances (toxic, hazardous, and dangerous chemicals and oil) and pollution to surrounding water quality caused by a chain reaction of traffic accidents. The “4.25 event” accords with the characteristics of the maximum credible accident. The dynamic fuzzy comprehensive classification algorithm is selected to classify the “4.25” WEE. The events at the same level are further divided by cluster analysis and minimum distance method to improve the scientific value and rationality of EDM.

In this work, the classification index and weight of WEE are applied by a questionnaire survey with nine grading indexes. The various indexes include nature of the leakage substances, treatment of leakage substances, total amount of leakage substances, emergency time, emergency duration, emergency form, leakage location, water flow velocity, and wind speed. These indexes are scored by five-point scale criteria. The Euclidean distance is calculated by comparing the classification standard level in the WEE classification database.

Each grading index is scored, and finally the comprehensive score is 3.366 by the five-point scale method. The cluster analysis result with the case information in the database indicated that the “4.25 event” belongs to the first level of WEE, and the corresponding emergency response should be implemented.

The next case analysis and deduction only need to retrieve scenarios and disposal plans corresponding to the first type of water environmental emergencies. Then, the case with high similarity in the case base of this level is searched to generate an emergency disposal plan.

### 4.5. Scenario Analysis

Scenario attribute of the “4.25 event” EDM based on scenario SR is determined before calculating the similarity between the “4.25 event” real scenario and the case scenario.

#### 4.5.1. Node Variable Determination and Network Construction

The scenario network of the “4.25 event” is developed from disaster, disaster-resistant, and disaster-bearing bodies based on scenario 3D representation of WEE. In summary, the scenario network node variables and values of the “4.25 event” are shown in Table 6.

The similarity satisfies the retrieval requirement by choosing reliability threshold u = 0.85. The process of determining the Bayesian network structure relationship is demonstrated by the determination of network causality between the “pollutant leakage quantity Y_3_” node and the remaining nodes. The basic probability assignment function m_i_ (T, F) of the causality between Y_3_ and other variables is scored by five experts. In the function, T means that causality exists between the two variables, while F indicates that no direct causality is present between the two variables. Only the probability assignment function, which the experts regard as a direct causality between the two variables, is listed in the table. The combination m (T) of the causality between Y3 and other variables in the disaster-bearing body dimension is calculated. The causality is established if m (T) is ≥0.85. Calculation results are shown in Table 7.

**Figure 6:**
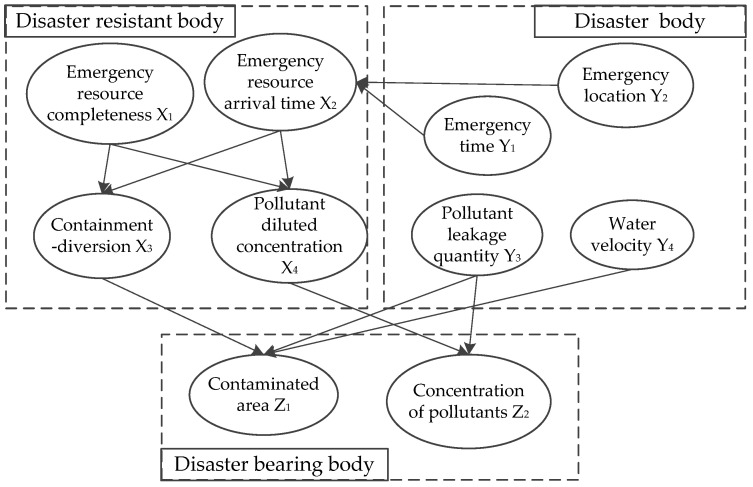
Bayesian network structure for scenario analysis of WEE.

Accordingly, m (T) = 0.21 ≤ 0.85 in Z_3_, so the causality of Z_3_ is unestablished, and the variable is rejected. Therefore, disaster-bearing body dimension only exhibits two variables, namely, contaminated area Z_1_ and concentration of pollutants Z_2_. The relationships among the node variables of WEE can be calculated through the above-mentioned method. Then, the Bayesian network structure of WEE scenario analysis can be obtained, as shown in Figure 6.

The values of the known variables, namely, X_1_, X_2_, Y_1_, Y_2_, Y_3_, and Y_4_, can be calculated by D–S evidence theory. Accordingly, the Bayesian network structure is obtained. As shown in Figure 6, emergency resource completeness X_1_ will affect containment-diversion X_3_ and pollutant diluted concentration X_4_. Then, the effect of X_3_ will further influence contaminated area Z_1_. This occurrence is consistent with the actual situation wherein the contaminated area is greatly diminished when the effect of containment-diversion is satisfied. Moreover, the contaminated area is expanded when the effect is non-ideal.

#### 4.5.2. Scenario Network Deduction

The Bayesian network structure and conditional probability of all nodes are inputted to calculation software Hugin Lite 8.1 (HUGIN EXPERT Company; Aalborg, Denmark), and then the probability value of each node variable are obtained by running the software program.

When obtaining evidence information, emergency time is in day, emergency location is far, pollutant leakage quantity is between 325 kg and 975 kg, water velocity is >1.5 m/s, and emergency resource completeness is adequate. The 3D node variable probability can be calculated, as shown in Table 8.

### 4.6. CBR

Scenario representation and case reasoning of the “4.25 event” are carried out according to scenario analysis-based CBR on the basis of scenario analysis. The principle of emergency plan selection is proven.

#### 4.6.1. XML-Based WEE Case Scenario Representation

The main feature attributes of the “4.25 event” scenarios are obtained in accordance with the collection of WEE cases and above-mentioned content of scenario knowledge representation.

#### 4.6.2. CBR of WEE Based on SR

The retrieved feature attributes of the “4.25 event” and case scenarios are shown in Table 9. The similarity calculation is performed according to the above-mentioned similarity algorithm between “4.25 event” and case scenarios S_1_ and S_2_. The calculation example of $\mathrm{sim}\left(s_{0_{i}},s_{1_{i}}\right)$ and $\mathrm{sim}\left(s_{0_{i}},s_{2_{i}}\right)$ is followed.

Emergency resource completeness:

$$\mathrm{sim}\left(s_{0_{2}},s_{1_{2}}\right)=1-\frac{\left(2\times 0.7-0.6+0.4\right)/2}{1-0}=0.4$$

$$\mathrm{sim}\left(s_{0_{2}},s_{2_{2}}\right)=1-\frac{\frac{{\left(0.6-0.45\right)}^{2}+{\left(0.4-0.45\right)}^{2}}{2\;\times \;0.2}}{1-0}=0.9375$$

Emergency location:

$$\mathrm{sim}\left(s_{o_{5}},s_{1_{5}}\right)=1-\frac{0.6-0.5}{1-0}=0.9$$

$$\mathrm{sim}\left(s_{o_{5}},s_{2_{5}}\right)=1-\frac{0.7-0.5}{1-0}=0.8$$

The similarity of each retrieved feature attribute is multiplied by the corresponding weight, which is the global similarity between the emergency and the case scenarios. The weights are excluded in the scope of this study. Therefore, the weights are considered to be the same value as 1/9, and the results are: $\mathrm{sim}\left(s_{0_{i}},s_{1_{i}}\right)=0.73$, $\mathrm{sim}\left(s_{0_{i}},s_{2_{i}}\right)=0.62$. Therefore, case scenario S_1_ is more relevant to “4.25 event” scenario than case scenario S_2_. Emergency plan (F_s1_) and emergency response (J_s1_) of case scenario S_1_ is regarded as the basis for real-time EDM plan. From seven industry experts, the method is well suited to practical cases.

## 5. Discussion

Water environmental emergencies in expressway region exhibit typical characteristics, such as unconventionality, low frequency, evident specificity, and great harm. The emergency plan generation method based on conventional plan management demonstrates difficulties in meeting the need of EDM for such emergencies. This paper is devoted to solving the problem of EDM of water environmental emergencies in expressway region, and an emergency decision-making model is constructed by combining CBR and SR methods. This work improves the former model based on previous studies, proposes risk source identification and dynamic fuzzy comprehensive clustering classification of emergencies, and introduces it to the field of WEE in expressway region. Risk source identification, dynamic classification, EDM plan generated with scenario-based case reasoning, and EDM support system are implemented in this work to timely generate effective EDM plan.

The following results are discussed based on theoretical research and case analysis:

(1) Based on literature analysis, there is a lack of EDM research on water environment in expressway region and a lack of risk source identification and emergency classification in EDM. Compared with previous studies, this research introduces risk source identification and emergency classification mechanism, which can identify the categories of emergencies more quickly and provide more accurate data for subsequent calculations. The research also has a case base and case study processes, in order to ensure the system is sustainable and the efficiency of scenario retrieval is improved. But water environmental emergencies have a certain degree of rarity and lack plenty of historical data to be verified. The prior probability needs to be assigned by experts and the error can only be reduced but not eliminated. This will affect the causality and influence the degree of scenario elements and cause errors in scenario deduction. There may be mismatches between S_0_ and case scenarios in CBR, resulting in wrong results. However, due to the consistency of Bayesian posterior distribution, the reasoning will rely on the prior distribution less and less and the results will be more and more accurate with the increasing sample size.

(2) Through questionnaires, interviews and expert scoring, this study got identification of potential risk events and the most credible accident grading indicators of water environment emergency in expressway region. When an emergency occurs, emergency categories and levels can be immediately identified in the light of risk source identification list, event list, and dynamic classification of large credible incidents to subsequently provide basic information for EDM plan generation. Risk sources may have influences on each other and need further researches to complete the model. Treatment of different toxicities of hazardous chemicals should be serious in case of secondary accidents. Classifying hazardous chemicals, formulating detailed measures to deal with actual situations, and improving emergency disposal plans are recommended further.

(3) The conceptual model and method were established based on scenario analysis and CBR. For different expressway water environmental emergencies, if the risk source type changes, the method in this research can still apply to it. However, this study is based on the assumption that the risk sources are independent of each other. The research model needs to be adjusted when the mutual influence relationship between the risk sources is considered. In addition, if the scenario elements are not disaster body, disaster-resistant body, and disaster-bearing body, but disaster factors, hazard-affected body, disaster-gestating environment or others, the three-dimensional structure of water environmental emergency scenario elements may change.

(4) In the validation of method with a case study, we listed five main potential impacts on the water environment in “4.25” event and classify it, finally concluded that it belongs to the first level of WEE. We can calculate the results of node variables and generate Bayesian network structure to get the influence relationship between variables and obtain the 3D node variable probability. At last, by calculating similarity between “4.25 events” S_0_ and case S_1_, S_2_, we can make a conclusion that emergency plan (F_s1_) and emergency response (J_s1_) of case scenario S_1_ is regarded as the basis for real-time EDM plan. Eventually, the emergency disposal plan of “4.25 event” scenario S_0_ contains information reports, on-site disposal, established field command team, equipment security, personnel protection, hazardous chemical disposal, vehicle towing, on-site cleaning, and water intake emergency treatment. This study only uses one case to verify the applicability of the model, which still needs to be further improved and validated by more cases of different sizes. And although the EDM support system plays a certain role in the case study, its function is still to be fully developed and to be applied by more case. For instance, (1) the case base still needs further classification and supplementation, and (2) the case learning algorithm needs to be developed. This task is difficult and requires a substantial amount of energy and time, but it also has profound significance. Scientific and applicable learning rules can improve the efficiency and accuracy of EDM, shorten emergency response time, achieve rational allocation of resources, and minimize the harm of WEE.

## 6. Conclusions

This study focused on the emergency decision-making for the water environmental emergencies in the expressway region, which aims to provide help for timely and effective generation of emergency plans to avoid serious environmental pollution and minimize various economic losses. The method proposed in this paper combines emergency classification with emergency decision-making and constructs an EDM model based on SR and CBR. Compared with the previous emergency decision-making models, our model is more targeted and practical in solving the problem of water environment emergency in expressway region. On the one hand, this study introduced risk source identification and emergency classification into the existing model, and obtained the indicators of risk source and event classification through questionnaires, interviews and expert scoring. On the other hand, this study used scenario analysis and CBR to establish a conceptual model. The model and methods were applied to a water environment emergency “4.25 event” in the DG expressway project to verify its validity. The study contributes to EDM in expressway water environment during the implementation stage, and provide a deeper insight to the development of a decision support system for emergency management.

Nevertheless, the study was still limited in terms of methods and model. These aspects in the EDM field can be further studied in the future: firstly, implicit and intrinsic emotional information requires an in-depth study in the scenario analysis and deduction to solve specific events. Because for specific emergencies, the scenario information includes not only explicit, external things or environment, but also some hidden and intrinsic emotional information, such as the psychological state of the affected population. Secondly, the EDM support system need be further developed through emergency management practices, such as supplementing the case base, studying the case learning algorithm and improving the case learning mechanism. 

## Figures and Tables

**Table 1:** Questionnaire results statistics.

Ordinal	Potential Risk Events	Mean	S.D
1	The water blocking belt is not designed or designed deficiently, and toxic or harmful leaking substances caused by emergencies can not be stored	4.53	0.622
2	The double drainage collection system is not designed or designed deficiently, and the bridge deck rain water can not be fully collected	4.07	0.704
3	The double protective fence is not designed or designed deficiently, the vehicle capsized due to vehicle collision could not be cushioned	4.25	0.887
4	Improper selection of construction materials, such as asphalt pavement is toxic and not resistant to wear, and the wearing substance is washed with heavy rain to form road surface runoff	4.33	0.684
5	The accident emergency pool is not designed or designed deficiently, and it can’t deal with the dangerous goods leaked from the emergency effectively	4.35	0.744
6	Design of water environmental protection area and marking	3.88	0.885
7	The construction of bridge foundation interferes with groundwater and affects the quality of surface water or causes groundwater, surface water runoff pollution	4.16	0.839
8	Oil leakage occurred in mechanical equipment, which was not treated in time, resulting in water quality pollution	4.23	0.847
9	When the bridge is constructed across the water body, the measures to prevent the project waste from falling into the water body are not taken	3.99	0.923
10	Arbitrary discharge of domestic sewage and garbage from staff	4.00	0.930
11	Leakage of construction material transportation	4.12	0.958
12	The design of the sedimentation treatment tank of construction wastewater is deficient, so that the construction wastewater is discharged into the water without effective treatment	4.28	0.763
13	During the construction period, temporary construction sites such as construction camps, prefabricated parts yards and stacking yards are set up within the scope of drinking water environmental protection areas	4.00	0.915
14	Burning and explosion accidents occur in warehouses where dangerous, toxic and flammable materials are stored	4.62	0.583
15	Warning signs are set up in the area of expressway water environment protection area to remind drivers of vehicles containing dangerous goods and oil	4.41	0.660
16	Rollover of vehicles carrying toxic and hazardous chemicals and oils cause leakage.	4.68	0.596
17	Leakage caused by vehicles carrying toxic and harmful chemical dangerous goods and oil falling into the water	4.71	0.540

**Table 2:** Cranbach Alpha coefficient of the questionnaire results of potential risk events.

Reliability Statistics		
Cronbach’s Alpha	Cranbach Alpha Based on Standardized Items	Number of Items
0.889	0.879	17

**Table 3:** Affecting factors and grading indicators study result.

Ordinal	Evaluation Indicator	Mean	S.D
1	Leakage substances	4.84	0.404
2	Treatment of leakage substances	4.56	0.620
3	Total amount of leakage substances	4.51	0.578
4	Emergency time	4.15	0.692
5	Emergency duration	4.39	0.676
6	Emergency form	4.35	0.596
7	Leakage location	4.55	0.599
8	Water flow velocity	4.12	0.915
9	Wind speed	3.88	1.038

**Table 4:** Cranbach Alpha coefficient of the survey results of evaluation indicators.

Reliability Statistics		
Cronbach’s Alpha	Cranbach Alpha Based on Standardized Items	Number of Items
0.747	0.747	9

**Table 5:** Risk source identification list.

Ordinal	Emergency	Potential Impacts on Water Environment
1	Asphalt pavement	Hazardous substances flow into surrounding water with runoff discharge
2	Damaged pavement runoff collecting system	Surface runoff directly scours the ground and enters the surrounding water body or underground water
3	Improper location of Rain Water treatment Station	All collected runoff and toxic and hazardous substances tip over into the water body
4	Domestic water and refuse discharge	Pollution of water body
5	Dangerous goods traffic accident	Dangerous goods enter the water body, pollute the river and affect water using safety

**Table 6:** Scenario network node variables and values of “4.25 event”.

Dimension	Index	Value Range and Quantization Score
Disaster- -resistant body	Emergency resource completeness X_1_	Scarce (0–0.3)/basically met (0.3–0.8)/adequate (0.8–1)
	Emergency resource arrival time X_2_	Relatively delayed (0–0.3)/relatively timely (0.3–0.8)/timely (0.8–1)
	Containment-diversion X_3_	Poor (0–0.3)/general (0.3–0.8)/satisfying (0.8–1)
	Pollutant diluted concentration X_4_	Pollutant concentration diminished by less than 30%/30% to 80%/more than 80%
Disaster body	Emergency time Y_1_	Day (1)/night (0)
	Emergency location Y_2_	Far (0–0.3)/moderate (0.3–0.8)/close (0.8–1)
	Pollutant leakage quantity Y_3_	Within the critical value (<325 kg)/ /one to threefold the critical value (≥325 kg and <975 kg)/more than threefold the critical value (≥975 kg)
	Water velocity Y_4_	<1.5 and >1.5 m/s
Disaster- -bearing body	Contaminated area Z_1_	Small area pollution (0–0.3)/large area pollution (0.3–0.8)/basically total pollution (0.8–1)
	Concentration of pollutants Z_2_	<3/≥3, and <10/≥10 mg/L
	Time of arrival at the water intake Z_3_	Short (<5 min)/moderate (≥5 and <40 min)/long (≥40 min)

**Table 7:** Calculation results of node variables.

Causality		m_1_	m_2_	m_3_	m_4_	m_5_	m (T)
Y_3_	Z_1_	0.90	0.80	0.70	0.85	0.95	0.95
	Z_2_	0.99	0.95	0.96	0.94	0.90	0.99
	Z_3_	0.69	0.50	0.58	0.64	0.75	0.21

**Table 8:** State probability of each node variable in WEE scenario.

Dimension	Node Variable	Value Range	State Probability
Disaster body	Emergency time	Day (1)/night (0)	(1, 0) evidence information
	Emergency location	Far/moderate/close	(1, 0, 0) evidence information
	Pollutant leakage quantity	<325/≥325 and <975/≥975 kg	(0, 1, 0) evidence information
	Water velocity	≤1.5 and >1.5 m/s	(1, 0, 0) evidence information
Disaster- -resistant body	Emergency resource completeness	Scarce/basically met/adequate	(0, 1, 0) evidence information
	Emergency resource arrival time	Relatively delayed/relatively timely/timely	(0.27, 0.52, 0.21)
	Containment-diversion	Poor/general/satisfying	(0.26, 0.50, 0.24)
	Pollutant diluted concentration	Less than 30%/30% to 80%/more than 80%	(0.29, 0.52, 0.19)
Disaster- -bearing body	Contaminated area	Small area pollution/large area pollution/basically total pollution	(0.65, 0.24, 0.11)
	Concentration of pollutants	<3/≥3, and <10/≥10 mg/L	(0.34, 0.46, 0.19)

The analysis result of the actual scenario indicated that the deduction result is consistent with the “4.25 event”. This finding indicates that the Bayesian network-based WEE scenario analysis method is feasible and effective.

**Table 9:** Calculation results of similarity between “4.25 events” S_0_ and case S_1_, S_2_.

Attribute	“4.25” Scenario S_0_	Case Scenario S_1_	Case Scenario S_2_	$sim\left(s_{0_{i}},s_{1_{i}}\right)$	$sim\left(s_{0_{i}},s_{2_{i}}\right)$
Pollutant type	oil spilling	oil spilling	oil spilling	0	0
Emergency resource completeness	0.4–0.6	0.7	0.45	0.4	0.9375
Emergency resource arrival time	0.3–0.6	0.5	0.6	0.92	0.85
Emergency time	night	night	day	1	0
Emergency location	0.5	0.6	0.7	0.9	0.8
Pollutant leakage quantity	400–600	700	660	0.7	0.72
Water velocity	1.2 m/s	1.1 m/s	1.4 m/s	0.97	0.93
Contaminated area	0.4–0.6	0.65	0.70	0.725	0.7
Concentration of pollutants	6–8	7	9	0.975	0.6

## Data Availability

Data is contained within the article.
